# A Tissue Culture Model of Murine Gammaherpesvirus Replication Reveals Roles for the Viral Cyclin in Both Virus Replication and Egress from Infected Cells

**DOI:** 10.1371/journal.pone.0093871

**Published:** 2014-04-02

**Authors:** Francine M. Scott, Samuel H. Speck

**Affiliations:** 1 Department of Microbiology and Immunology, Emory University School of Medicine, Atlanta, Georgia, United States of America; 2 Emory Vaccine Center, Emory University School of Medicine, Atlanta, Georgia, United States of America; Geisel School of Medicine at Dartmouth, United States of America

## Abstract

Passage through the eukaryotic cell cycle is regulated by the activity of cyclins and their cyclin-dependent kinase partners. Rhadinoviruses, such as Kaposi’s sarcoma-associated herpesvirus (KSHV) and murine gammaherpesvirus 68 (MHV68), encode a viral homologue of mammalian D-type cyclins. In MHV68, the interaction of the viral cyclin with its CDK partners is important for acute replication in the lungs following low dose inoculation. Attempts to further study this requirement *in vitro* have been limited by the lack of available tissue culture models that mimic the growth defect observed *in vivo*. It is hypothesized that analysis of virus replication in a cell line that displays properties of primary airway epithelium, such as the ability to polarize, might provide a suitable environment to characterize the role of the v-cyclin in virus replication. We report here MHV68 replication in the rat lung cell line RL-65, a non-transformed polarizable epithelial cell line. These analyses reveal a role for the v-cyclin in both virus replication, as well as virus egress from infected cells. As observed for acute replication in vivo, efficient replication in RL-65 cells requires CDK binding. However, we show that the KSHV v-cyclin (K-cyclin), which utilizes different CDK partners (CDK4 and CDK6) than the MHV68 v-cyclin (CDK2 and CDC2), can partially rescue the replication defect observed with a v-cyclin null mutant – both in vitro and in vivo. Finally, we show that MHV68 is shed from both the apical and basolateral surfaces of polarized RL-65 cells. In summary, the RL-65 cell line provides an attractive in vitro model that mimics critical aspects of MHV68 replication in the lungs.

## Introduction

The eukaryotic cell cycle is a tightly regulated and sequentially executed pathway by which DNA is duplicated and partitioned into daughter cells. Cyclins and their cyclin-dependant kinases (CDKs) form active complexes that facilitate this progression. Each phase of the cell cycle is associated with its own unique cyclin activity and control mechanisms. Low RNA and protein synthesis occurs in quiescent cells, and this state is referred to as G_0._ It is upon mitogenic stimulation that many of these cells upregulate cyclin D which partners with CDK4 and/or 6 to drive entry into the G1 phase of the cell cycle. Cell growth, phosphorylation of retinoblastoma protein (pRb), cyclin E synthesis, and factors required for DNA synthesis and passage through the restriction point are all properties of the G1 phase [1]. Entry into the next phase of the cycle, S phase, is driven by cyclin E. Cyclin E interacts with CDK2 to drive E2F transcriptional activity which pushes the cell farther into the cycle and drives the production of yet another cyclin, cyclin A. Cyclin A also partners with CDK2 and will promote DNA replication and progression through S phase, as well as entry into the G2 phase of the cell cycle. The early events of mitosis occur during G2, which include cyclin B:CDK1 activity that facilitates full progression into mitosis. Eukaryotic cells also employ mechanisms to regulate cyclin:CDK activities. Catalytic subunit inhibition and cyclin:CDK assembly promotion by CKIs are just two examples of how the cell cycle can be kept in check [2].

Although the eukaryotic cell cycle is very tightly controlled, this has not stopped many viruses from exploiting various steps in this pathway to create a cellular environment conducive for their replication. Gammaherpesviruses, which include Epstein-Barr virus (EBV), Kaposi’s sarcoma-associated herpesvirus (KSHV) and murine gammerherpesvirus 68 (MHV68), are just one example of a virus subfamily that has evolved strategies for modulating the host cell cycle in their favor. For example, the rhadinovirus subgroup of the gammaherpesvirus family (e.g., KSHV and MHV68) encode a viral homologue of eukaryotic D type cyclins. These homologues all share conserved residues with D type cyclins, most prominently in the cyclin box – a domain that is critical for CDK binding [3]. The KSHV viral cyclin (K-cyclin) associates predominately with cellular CDK 6, but can also interact with CDKs 2, 4, 5, and 9 [4], [5]. The MHV68 viral cyclin has been shown to preferentially interact with cellular CDK2 and CDC2/CDK1 [6]. Unlike their eukaryotic counterparts, both KSHV and MHV68 v-cyclins are resistant to the action of some CKIs and thus can evade normal host cell control [6], [7]. Despite encoding these specific mechanisms to manipulate the host cell cycle, much of how this modulation of cyclin activity is directly connected with viral pathogenesis remains to be elucidated. The species specificity of the human rhadinovirus KSHV makes *in vivo* studies challenging, thus making the rodent model utilizing MHV68 infection of laboratory strains of mice an attractive alternative.

MHV68 is a natural pathogen of murid rodents that has been shown to establish chronic infections in in-bred strains of laboratory mice. Acute MHV68 infection following intranasal inoculation is characterized by productive infection in the lung, spleen, and liver. MHV68 establishes latency in professional antigen presenting cells: B cells, macrophages, and dendritic cells [8], [9]. Immune impairment of mice is correlated with various conditions after infection including fibrosis, vasulitis, or neurological disease [10]–[14]. In mice with healthy immune systems the primary pathology after MHV68 infection is interstitial pneumonia, which is largely cleared by days 9–12 post-infection [15].

The MHV68 viral cyclin homologue (v-cyclin) is an important regulator of reactivation from latency, replication in the lungs (at low dose) and, when expressed as a transgene, is a potent oncogene [16]–[18]. Generation of CDK binding mutants in the v-cyclin have shown that the viral cyclin:CDK interaction is necessary for the virus to replicate to WT levels in the lungs after low dose. However, those same CDK binding mutants, in contrast to the v-cyclin null virus, are able to reactivate from latency to near wild type virus levels - indicating a CDK-independent function of the v-cyclin important for virus reactivation [17]. In interferon-γ deficient mice on a BALB/c background the v-cyclin critically contributes to acute lethal pneumonia [19] and fibrosis of the lungs [20]. However, attempts to further study the importance of the MHV68 viral cyclin using tissue culture models have failed to reveal a role for v-cyclin [17]. One possible explanation for this disconnect between virus behavior in vivo and what is observed in vitro is postulated to be the differences in epithelial state between established cell lines and that of host lungs [17].

MHV68 infection via the intranasal route leads to viral engagement with only the surface, superficial epithelium, which is highly differentiated [21]. The process by which an epithelial cell becomes fully differentiated involves exit from the cell cycle, acquisition of epithelial specific molecular markers, and asymmetric separation of various cellular properties (polarization) [22], [23]. Many conventional epithelial cell lines are derived from a transformed progenitor and do not polarize; thus they can continue to cycle and do not take on many of the polarization properties inherent of airway epithelium. Therefore, it seems likely that one or more of the properties unique to differentiated epithelium are critical for an environment in which the function of the MHV68 cyclin D homologue becomes most apparent. Here we report analyses of MHV68 replication, and the role of the viral cyclin, utilizing an epithelial cell line (RL-65) that exhibits many properties of airway epithelium - including the ability to form polarized monolayers on transwells [24], [25].

## Results and Discussion

### MHV68 Requires the Viral Cyclin for Efficient Replication in RL-65 Epithelial Cells

**Figure 1:**
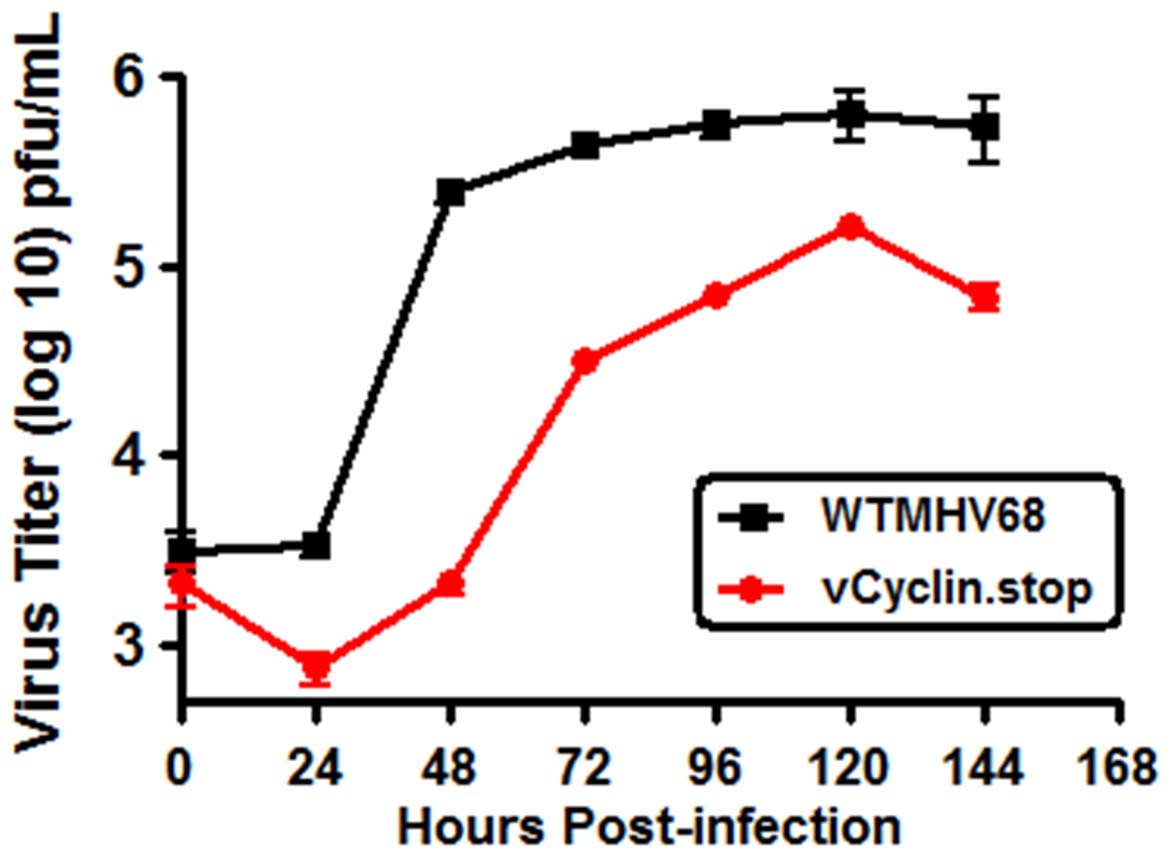
MHV68 requires v-cyclin function for robust growth at early time points in growth arrested RL-65 cells. RL-65 cells were plated to confluency and then infected at an MOI of 0.05 with either WT MHV68 or vCyclin.stop virus. Cells plus supernatants were collected every 24 hours and subjected to freeze/thaw twice to release any intracellular virus. Total virus at each time point was determined by plaque assays on NIH 3T12 fibroblasts. The data shown was compiled from 3 separate experiments, each done in triplicate. Standard error of the mean is shown. P values for the observed difference in the replication of WT MHV68 vs the vCyclin. Stop mutant, determined by an unpaired two-tail T-test at each time point, were: 24 hrs, p = 0.0084; 48 hrs, p = 0.0023; 72 hrs, p = 0.0006; 96 hrs, p = 0.0046; 120 hrs, p = 0.0612; and 144 hrs, p = 0.0779.

MHV68 requires the viral cyclin to replicate efficiently in the lungs of mice after low dose inoculation [17]. After screening a number of fibroblast and epithelial cell lines in which we failed to identify a significant replication defect of the v-cyclin null virus, we report here MHV68 growth in the rat lung cell line RL-65. RL-65 cells are a spontaneously immortalized, non-transformed epithelial cell line that was originally derived from neonatal rat lungs by careful manipulation of microenvironment to select for a cell type that maintained highly differentiated features *in vitro* [24]. They are propagated in media supplemented with growth factors rather than sera in order to more accurately mimic the surroundings the lung epithelium would be subjected to *in vivo* [24]. To address whether the RL-65 cell line would provide an environment in which the dependence on the v-cyclin in viral growth would be apparent, confluent monolayers of RL-65 epithelium were infected at an MOI of 0.05. The v-cyclin null virus (vCyclin.stop), which has been extensively characterized [16], [17], harbors a translation stop codon near the amino-terminus of the v-cyclin open reading frame. After infection of the monolayer, cells and supernatants were harvested every 24 hours for a total of 144 hours for analysis of total virus growth (Figure 1A). Notably, there was a significant defect in vCyclin.stop virus replication compared to WT MHV68 at early times post-infection (at 48 hrs post-infection there was a ca. 100-fold defect in virus replication). However, the vCyclin.stop virus was able to generate nearly equivalent virus titers as WT MHV68 by late time post-infection (Figure 1A,1B).

### The KSHV v-cyclin can Partially Rescue Replication of MHV68 Lacking a Functional v-cyclin, Both in vitro and in vivo

**Figure 2:**
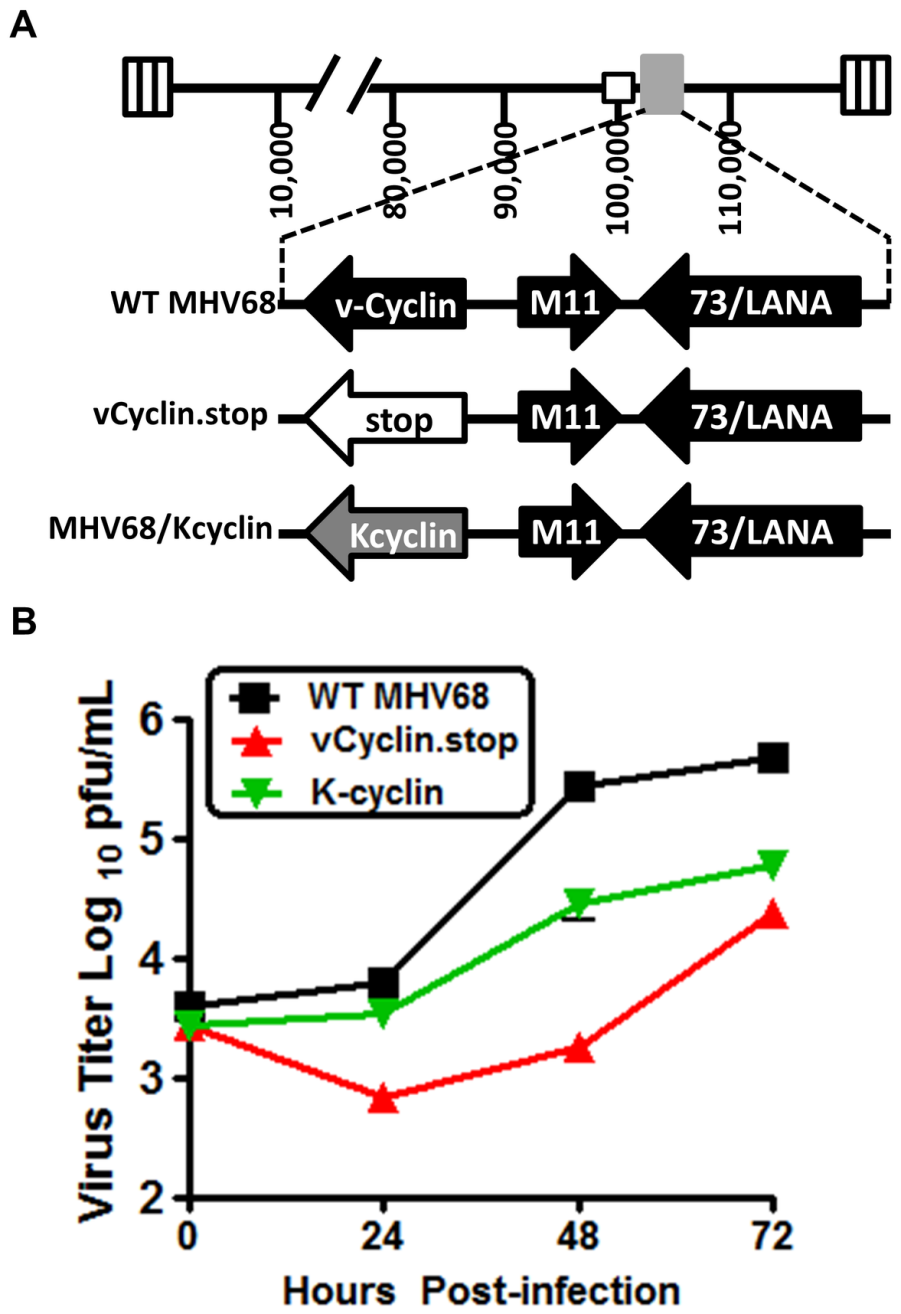
KSHV v-cyclin can partially rescue MHV68 v-cyclin null virus replication defect in growth arrested RL-65 cells. (A) Schematic illustration depicting the v-cyclin locus and insertion of KSHV v-cyclin in place of the MHV68 v-cyclin open reading frame. (B) RL-65 cells were plated to confluency and then infected at an MOI of 0.05 with either WT MHV68, vCyclin.stop or a recombinant MHV68 expressing the KSHV v-cyclin from the MHV68 v-cyclin locus (K-cyclin). Cells plus supernatants were collected every 24 hours and subjected to freeze/thaw twice to release any intracellular virus. Total virus at each time point was determined by plaque assays on NIH 3T12 fibroblasts. The data shown was compiled from 3 separate experiments, each done in triplicate. Standard error of the mean is shown.

**Figure 3:**
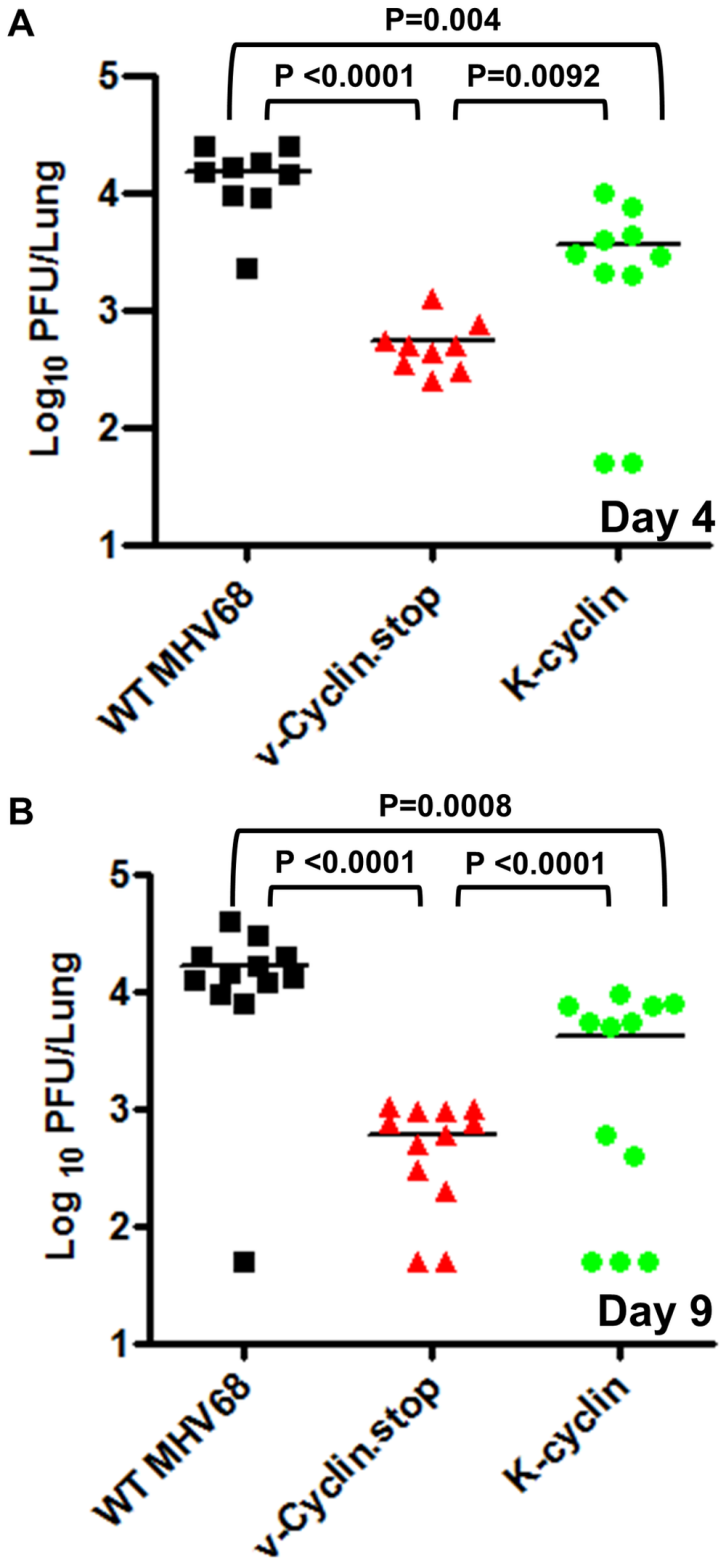
KSHV v-cyclin can partially rescue MHV68 v-cyclin null virus replication defect in lungs following intranasal inoculation. C57Bl/6 mice were infected via intranasal inoculation with 1,000 pfu of either WT MHV68, vCyclin.stop or a recombinant MHV68 expressing the KSHV v-cyclin from the MHV68 v-cyclin locus (K-cyclin). Lungs were harvested at days 4 (panel A) and 9 (panel B) post-infection, and total virus at each time point was determined by plaque assays on NIH 3T12 fibroblasts as described in Materials and Methods. Each symbol represents analysis of an individual infected mouse.

**Figure 4:**
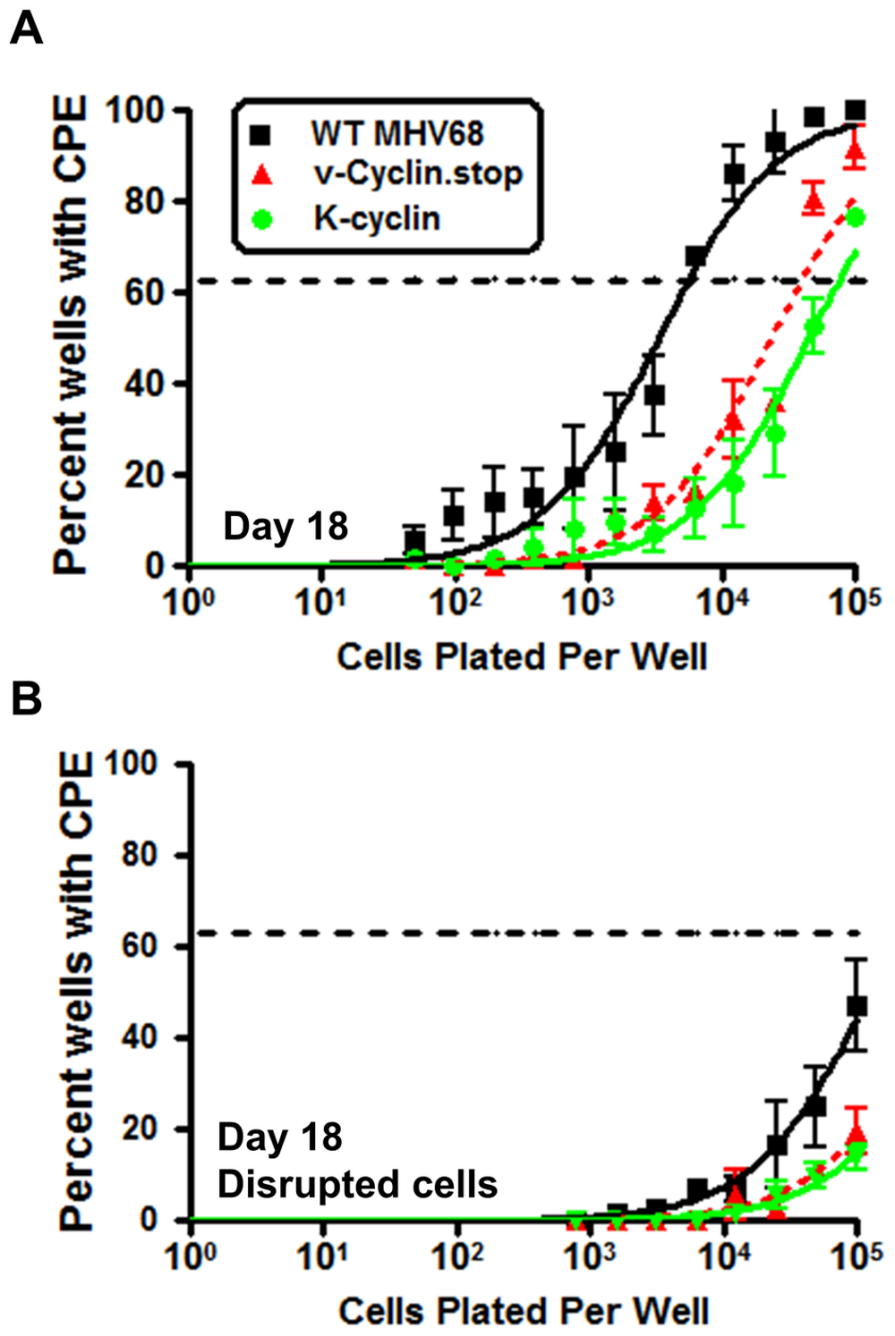
KSHV K-cyclin does not rescue MHV68 v-cyclin null virus reactivation from splenocytes following intranasal inoculation. C57Bl/6 mice were infected via intranasal inoculation with 1,000 pfu of either WT MHV68, vCyclin.stop or a recombinant MHV68 expressing the KSHV v-cyclin from the MHV68 v-cyclin locus (K-cyclin). (A) Reactivation from splenocytes on day 18 post infection was determined by assessing the percent of wells positive for viral CPE after limiting dilution onto MEF indicator monolayers as described in Materials and Methods. (B) Amount of preformed infectious virus was determined by assessing mechanically disrupted splenocytes. The analyses shown were compiled from 3 separate experiments, using 4 mice per experiment for each virus. Standard error of the mean is shown.

As discussed above, it is notable that the MHV68 and KSHV v-cyclins interact with different cellular CDKs, raising the question of whether this represents a divergence in their functions during virus infection. To begin to address this issue, we generated a recombinant MHV68 in which the v-cyclin open reading frame was replaced with the KSHV v-cyclin coding sequence. Upon low MOI infection of RL-65 cells the K-cyclin virus exhibited an intermediate phenotype in that it replicated faster at early times post-infection than the v-cyclin null mutant, but replicated more slowly than WT MHV68 (most apparent at 48 and 72 hrs post-infection) (Figure 2B). We extended this analysis to assess replication of the MHV68/K-cyclin virus in vivo. After intranasal inoclulation of C57Bl/6 mice with 1,000 pfu of either WT MHV68, vCyclin.stop or the MHV68/K-cyclin virus, lungs were harvested at days 4 and 9 post-infection. As observed in the *in vitro* growth analyses in RL-65 cells, the K-cyclin virus exhibited an intermediate phenotype *in vivo* (Figure 3A, 3B). These results suggest that either there is a function of MHV68 v-cyclin that is not recapitulated by the K-cyclin (which could reflect utilization of different CDK partners), and/or there is a technical issue related to regulation of K-cyclin expression from the MHV68 v-cyclin locus. Additionally, characterization of K-cyclin CDK interaction has been extensively characterized in human, monkey, and insectcells [4], [26]–[28]. Whether any differences in binding occur in cells from rodents is unclear, though the K-cyclin percent similarity is roughly identical (∼54%) when compared to mouse, human and rat D-type cyclins [26]. Regardless, these results demonstrate that the K-cyclin can significantly enhance replication of MHV68 in the absence of the MHV68 v-cyclin. To address whether K-cyclin enhancement of MHV68 replication in the absence of the MHV68 v-cyclin would rescue the v-cyclin null virus reactivation defect from splenocytes [17], we assessed virus reactivation at day 18 post-infection. Notably, the K-cyclin failed to increase the frequency of splenocytes reactivating virus at day 18 when compared to the vCyclin.stop mutant (Figure 4A, 4B). This indicates that although acute replication in the lungs is enhanced with the K-cyclin chimeric virus, this does not enhance virus reactivation from splenocytes – suggesting a function of the MHV68 v-cyclin involved in virus reactivation that is not conserved in the K-cyclin. Overall, the analyses of the vCyclin.stop and K-cyclin viruses in vivo, as well as in RL-65 cells, argue that replication in RL-65 cells accurately mimics the requirements for MHV68 replication in the lungs at the acute stage of virus infection.

### MHV68 is Shed from Both the Apical and Basolateral Surfaces of Polarized RL-65 Cells

**Figure 5:**
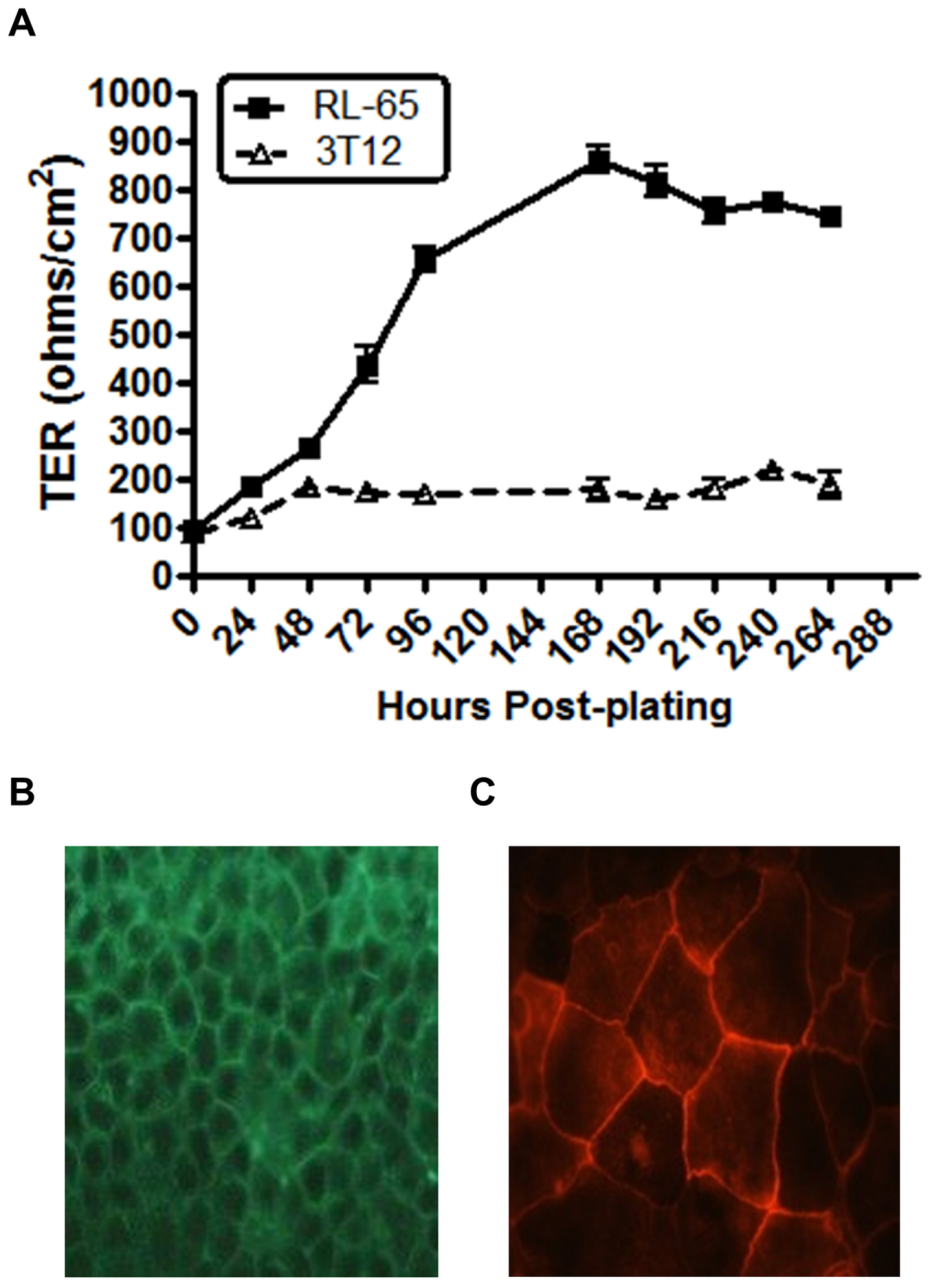
RL-65 cells form polarized monolayers in transwells. (A) RL-65 cells or, as a negative control, NIH 3T12 cells were plated at a concentration of 2×10^5^ cells/well onto 0.4 μM pore size transwell polyester membranes. Every 24 hours transepithelial resistance (TER) was measured. The RL-65 monolayer was infected with MHV68 (MOI = 0.05) at 168 hours post-plating, as indicated. The data shown was compiled from 3 separate experiments. Standard error of the mean is shown. (B & C) Confluent monolayers of RL-65 cells were stained for adherens junction marker E-cadherin (B) or tight junction marker zona occludens (C).

In polarized lung epithelium, the separation of the apical and basolateral surfaces by adhesive connections allows the tissue to form a distinct barrier between the outer surface and the lumen. In tissue culture models, the strength of this boundary can be measured by trans-epithelial resistance (TER). It has previously been shown that the RL-65 cell line can form high resistance epithelial monolayers when grown on transwell filters [25]. To confirm this, 2×10^5^cells were plated onto transwells and transepithelial resistance (TER) was measured every 24 hours. As the monolayer continued to grow, TER readings steadily increased to levels typical for this cell type (Figure 5A) [25]. As a negative control, NIH 3T12 fibroblasts were plated on transwell filters and TER measurements assessed over the same timeframe (Figure 5A). As expected, little increase in resistance was observed. Junctions between polarized epithelial tissues are composed of specific adhesion protein complexes; adherans junctions and tight junctions. To visualize the integrity of these junctions in RL-65 cells, confluent monolayers were stained for E-cadherin (adherans junction marker) and zona occludens 1 (tight junction marker) (Figure 5B, 5C). E-cadherin and zona occludens were visualized to localize predominately to the region between adjacent cells forming a “cobblestone” pattern. This is a strong visual indicator of the formation of intact junctions [25], [29]. Taken together, along with the high TER measurements, indicate properties of highly polarized epithelium.

**Figure 6:**
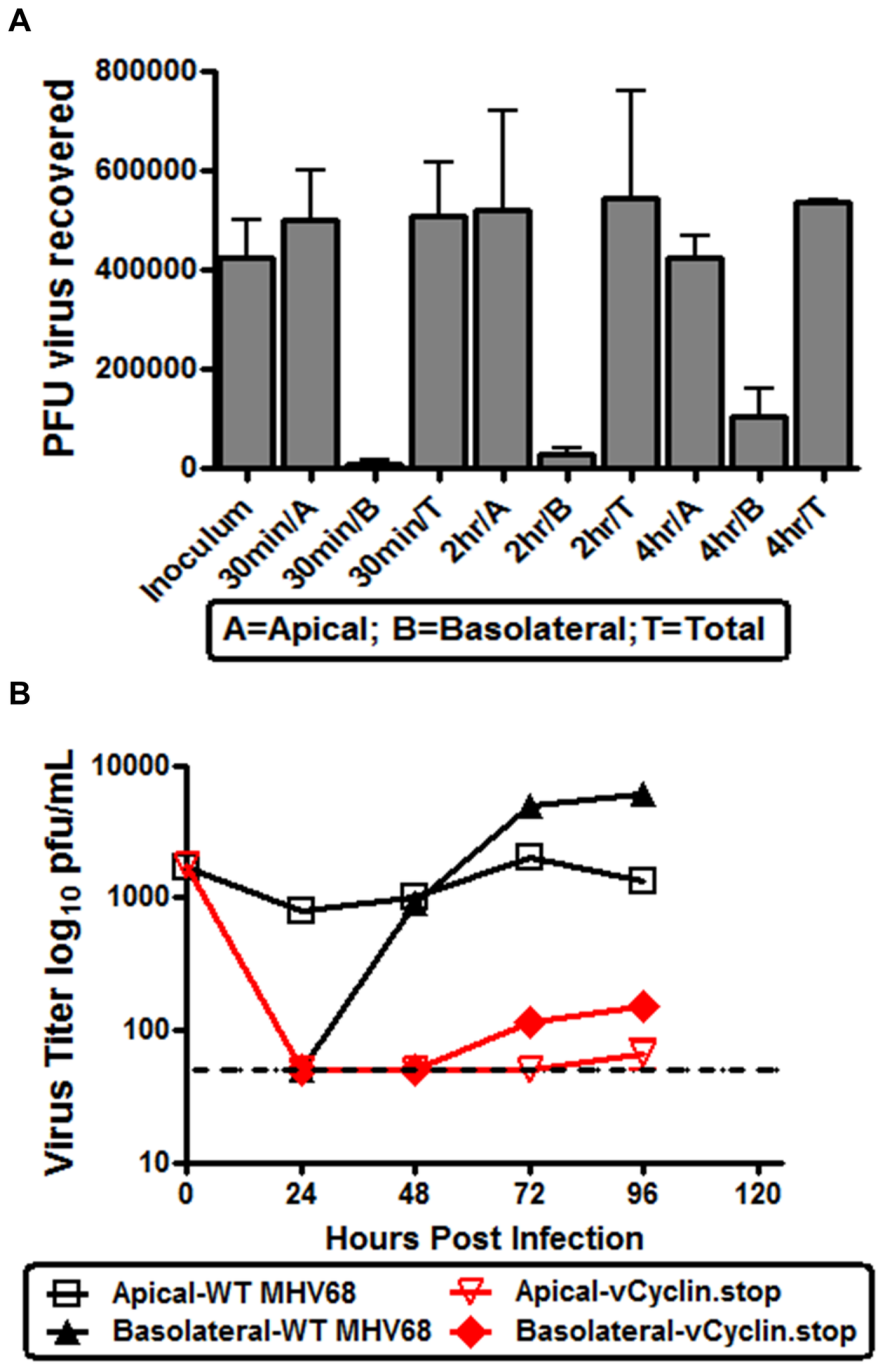
Basolateral shedding of MHV68. (A) RL-65 cells were plated at a concentration of 2×10^5^ cells per transwell. To assess virus diffusion, as well as potential virus trapping in transwell membranes, a known quantity of MHV68 (inoculum) was added to the apical chamber of empty transwells (no cells) (168 hrs post-plating cells). Samples from the apical and basolateral chambers were collected at various time points to determine number amount of virus present. Total virus reflects the sum of virus titers determined for the apical and basolateral chambers. (B) RL-65 cells were plated at a concentration of 2×10^5^ cells per transwell. Confluent monolayers of RL-65 cells (168 hours post plating) were infected at an MOI of 0.05 with WT or v.Cyclin.stop virus. Every 24 hours post-infection supernatant from the apical and basolateral chambers was collected and the amount of virus presented determined by plaque assay on NIH 3T12 fibroblasts. The dashed line indicates the limit of detection of the plaque assay. The data shown was compiled from 3 separate experiments, each done in triplicate. Standard error of the mean is shown.

Prior to characterizing MHV68 egress from polarized RL-65 cells, we assessed whether there was any binding of virus to transwell membranes as such binding might alter our interpretations of these analyses. As shown in figure 6A, when virus as added to empty transwells (i.e., in the absence of a cell monolayer) over a 4 hour timecourse the total amount of virus recovered was equivalent to the amount of input. Thus, there was no evidence of virus trapping/binding to the transwell membrane. To study the apical/basolateral egress of MHV68, RL-65 monolayer formation on the transwell membranes was monitored by TER every 24 hours as well as visual inspection. 168 hours post plating, confluent RL-65 cells were infected at an MOI of 0.05 with either MHV68 WT or vCyclin.stop virus apically. Every 24 hours, the supernatant was gathered from either the apical or basolateral chamber for quantification via plaque assay (Figure 6B). WT MHV68 was shed from both the apical and basolateral surfaces – with larger amount of virus being shed basolaterally at late times post-infection (Figure 6B). Notably, the v-cyclin null mutant showed a profound defect in egress from polarized RL-65 cells (Figure 6B). At 24 and 48 hours post-infection the amount of virus shed either apically or basolaterally was at or below the limit of detection (dotted line, Figure 6B). By 72 and 96 hrs post-infection a small amount of v-cyclin null virus was detectable and was mostly shed from basolateral surface of the polarized RL-65 cells.

**Figure 7:**
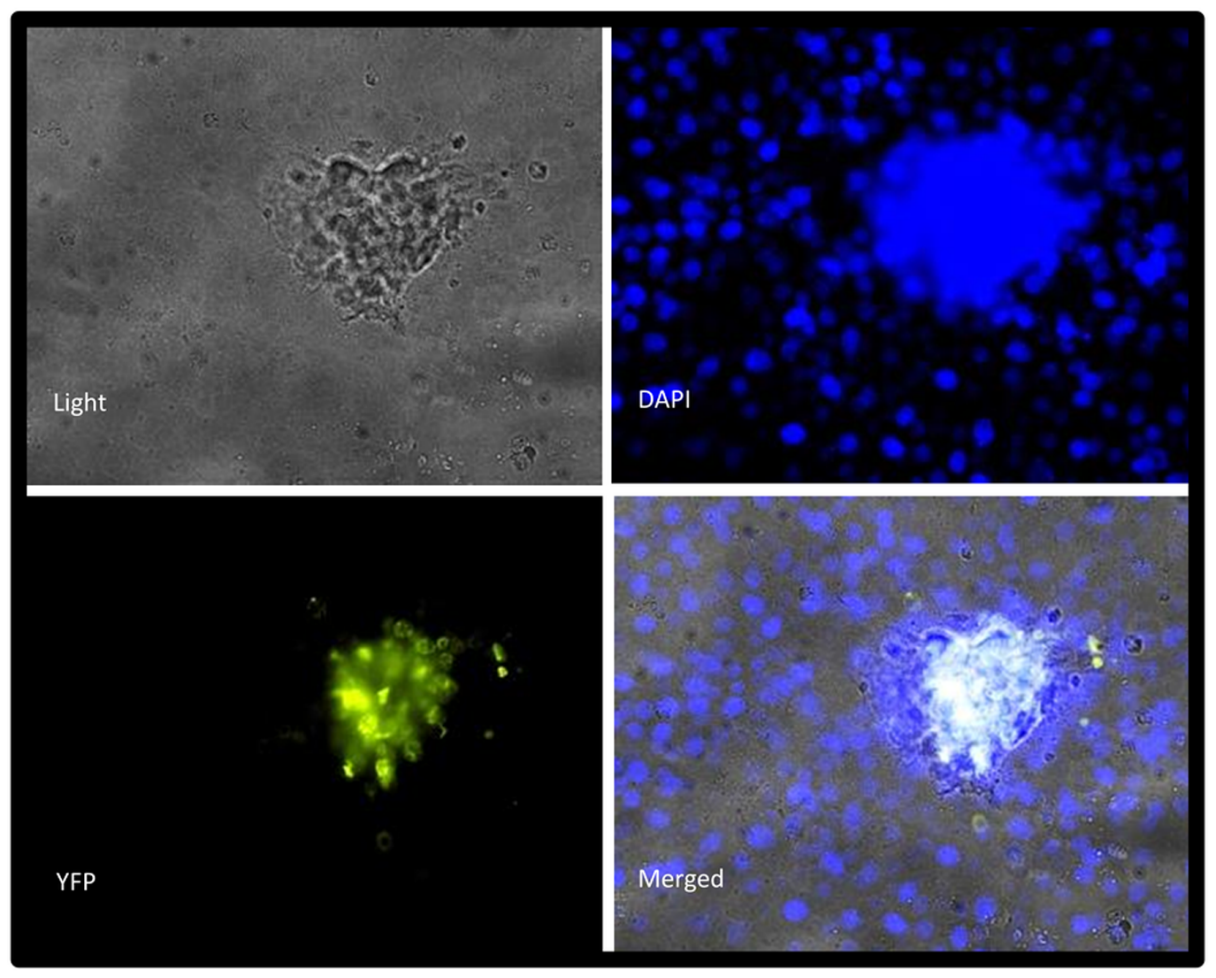
MHV68 infection of polarized RL-60 cells leads to piles of infected cells that appear to be extruded from the cell monolayer. (A) RL-65 cells plated to confluency onto glass chamberslides and infected at an MOI of 0.05 with MHV68-H2bYFP virus. 120 hours post-infection samples were fixed as described in Materials and Methods. Nuclei were stained with DAPI and virus infected cells detected by YFP expression.

TER measurements taken throughout the course of the experiment showed viral infection caused little change to the resistance of infected monolayer (Figure 5A), indicating MHV68 doesn’t significantly destroy the integrity of the epithelium during infection. However, during the course of all experiments in RL-65 cells it was observed that infection with MHV68 causes apparent changes in the morphology of the epithelium, with infected cells appearing to “pile” on top of each other and were visualized to be “out of plane” with the rest of the monolayer - yet never detaching from the monolayer, even up to 120 hours post-infection (Figure 7A, 7B and unpublished observations). It is unclear at this time whether the underlying epithelium is intact and whether this phenomenon influences the amount of virus that is detected in the apical and basolateral chambers. Future analyses of this phenomenon will benefit from the use of confocal microscopy/Z stack image acquisition for a 3-D visualization. However, we speculate that some of the apically shed virus arises from infected cells that are no longer part of the polarized epithelial monolayer and, as such, may confound the determination of amount of apically shed virus.

**Figure 8:**
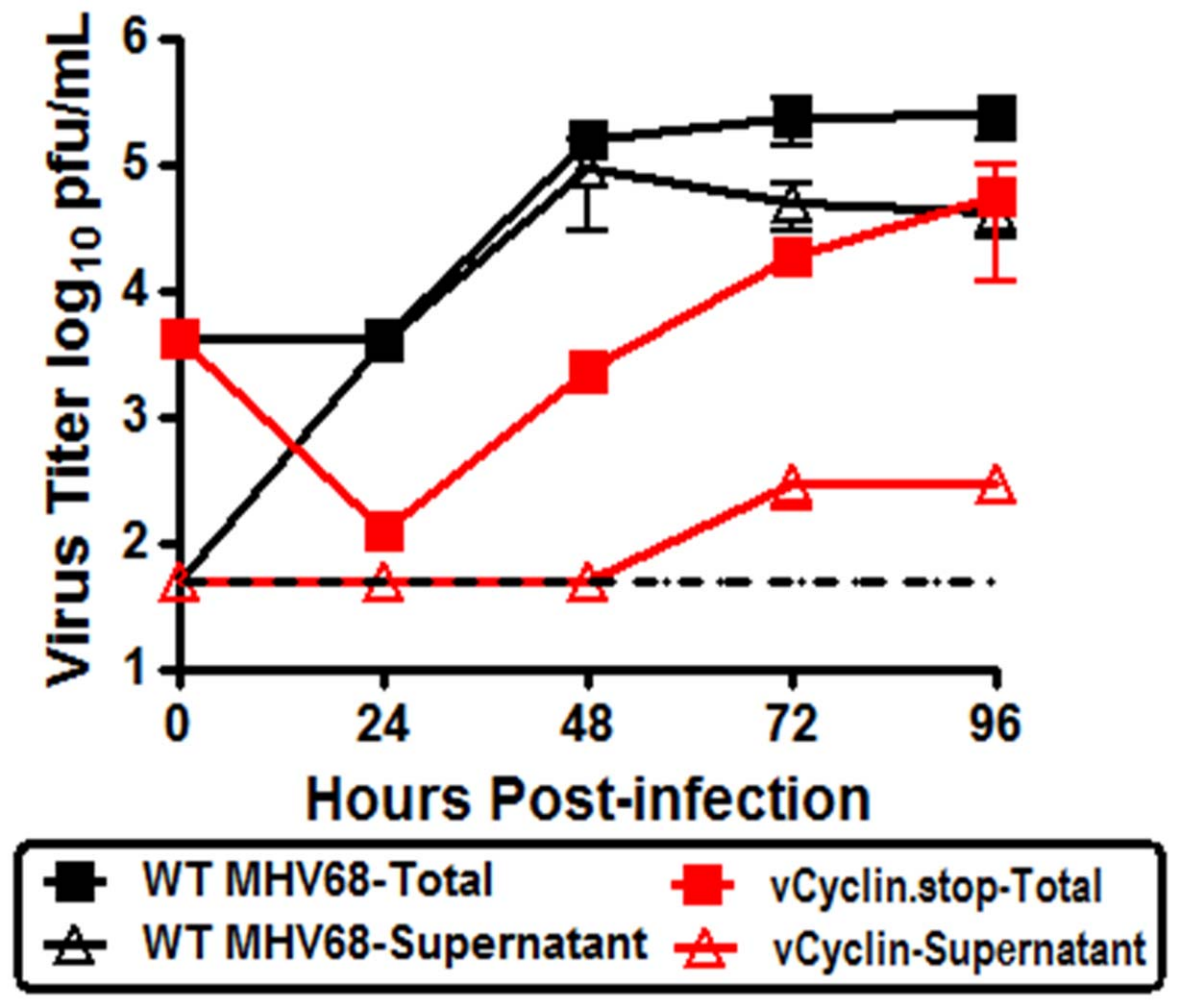
The vCyclin.stop mutant exhibits a severe defect in virus egress from growth arrested RL-65 cells. RL-65 cells were plated at a concentration of 2×10^5^ cells per transwell. Confluent monolayers were infected with an MOI of 0.05 with either MHV68 WT or vCyclin.stop. Every 24 hours either shed virus (supernatant from both apical and basolateral chambers) or total virus (cells plus supernatant from both apical and basolateral chambers) were collected for titer on NIH 3T12 fibroblasts. The dashed line indicates the limit of detection of the plaque assay. The data shown was compiled from 3 separate experiments, each done in triplicate. Standard error of the mean is shown.

The defect in egress of the v-cyclin null virus from polarized RL-65 cells was much greater than the defect in virus replication observed when total virus replication (cell associated plus released virus) was analyzed (compare Figures 1 and 6B). To further explore this issue, we repeated the analysis to compare total virus replication (cell associated plus released virus) to virus released from polarized RL-65 cells (Fig. 8). As previously observed, there was a significant defect in total virus replication which was most apparent are early times post-infection. However, when the amount of release virus was measure (apical plus basolateral), a much more profound defect was observed. Notably, the same analysis for WT MHV68 showed nearly equivalent levels of total and shed virus – indicating very efficient release of virus from polarized RL-65 cells (Figure 8). Thus, we conclude that the v-cyclin null virus has a profound defect in virus egress, as well as a defect in overall virus replication.

**Figure 9:**
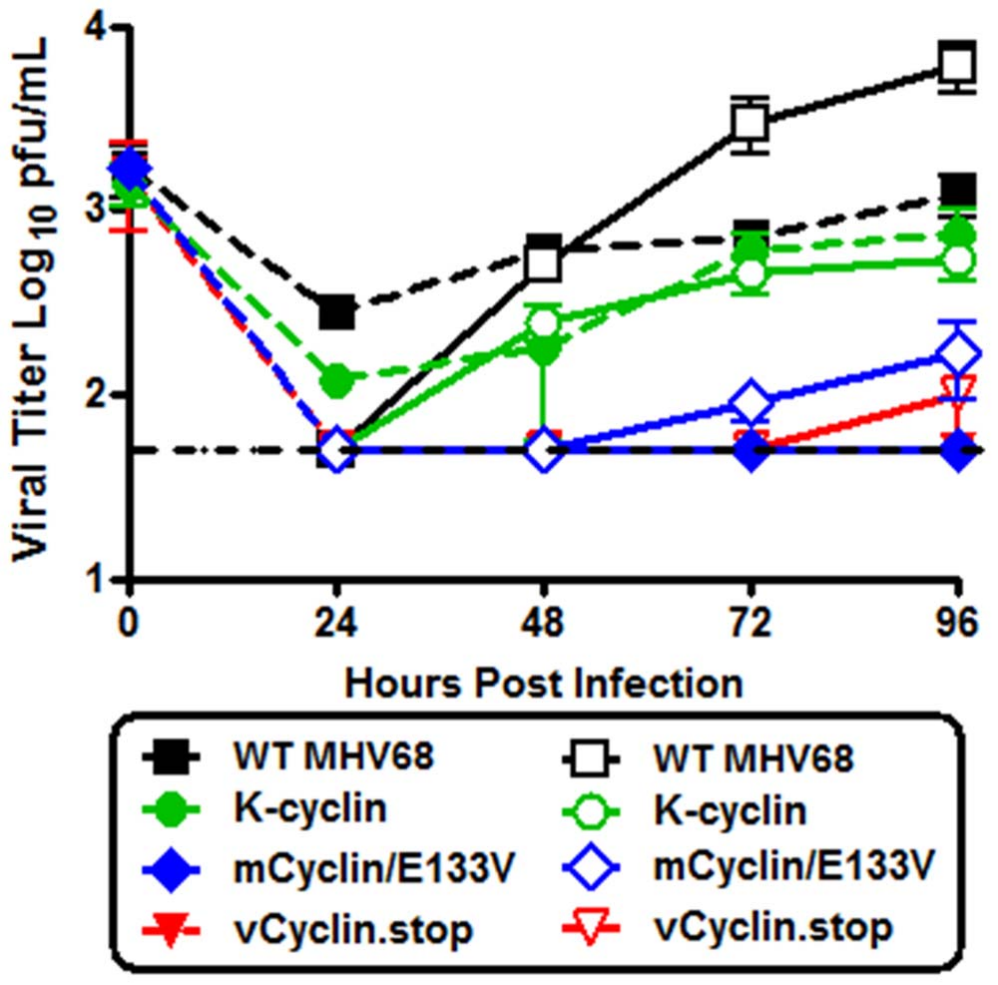
CDK binding is required for efficient MHV68 replication in RL-65 cells. Confluent monolayers of RL-65 cells (168 hours post plating) were infected at an MOI of 0.05 with the indicated viruses. Every 24 hours post-infection supernatant from the apical (closed symbols) and basolateral (open symbols) chambers was collected and the amount of virus presented determined by plaque assay on NIH 3T12 fibroblasts. The dashed line indicates the limit of detection of the plaque assay. The data shown was compiled from a single experiment done in triplicate. Standard error of the mean is shown.

Finally, because the role of the v-cyclin in acute MHV68 replication in the lungs of intranasally inoculated mice has previously been shown to be CDK-dependent, we compared egress of the K-cyclin, vCyclin.stop and a CDK binding mutant v-cyclin (mCyclin/E133V) from polarized RL-65 cells (Figure 9). Importantly, although the K-cyclin chimeric MHV68 exhibited a modest defect in virus replication (see Figure 2), it did not exhibit a defect in virus egress (Figure 9). In contrast, the CDK binding mutant v-cyclin (mCyclin/E133V) exhibited a similar egress phenotype to the v-cyclin null virus mutant (vCyclin.stop) (Figure 9). Thus, as expected the roles of the v-cyclin in virus replication and egress are both dependent on interaction with appropriate cellular CDKs. Other viruses, such as HCMV, have been shown to utlize the cyclin/CDK pathway in efficient dissassembly of the lamina for virus egress [30]. Additionally, many viruses remodel the host cytoskelton during egress [31], and CDK has been shown to be an important contributor to cellular remodeling [32], [33]. How specifically the MHV68 cyclin is involved in virus egress remains to be elucidated.

### Conclusions

The epithelium lining the lungs serves not only as a medium for gas exchange, but also as a barrier protecting the host from the outside environment. This lining is often only a single cell thick and its cells are polarized with distinct apical and basolateral domains [34]. Facing the lumen is the apical domain which characteristically contains properties to interact with the environment. The basolateral domain interfaces the underlying basement membrane, as well as cellular neighbors [34]. Separating these two surfaces are a variety of junction proteins that form strong connections between neighboring cells. It seems very likely that these cells serve as the initial barrier through which intranasally inoculated MHV68 must traverse to establish infection. Here we have shown that the characterization of MHV68 replication in a polarized, non-transformed lung epithelial cell line (RL-65) not only recapitulates the CDK-dependent role of the v-cyclin in virus replication, but also identifies a previously unknown role for the v-cyclin in egress from polarlized epithelium. Future studies will focus on the nature of the latter defect, which are likely to provide new insights into how MHV68 traffics in infected cells.

## Materials and Methods

### Ethics Statement

This study was carried out in strict accordance with the recommendations in the Guide for the Care and Use of Laboratory Animals of the National Institutes of Health. The protocol was approved by the Emory University Institutional Animal Care and Use Committee and in accordance with established guidelines and policies at Emory University School of Medicine (Protocol no. YER-2002245-031416GN).

### Tissue Culture

RL-65 cells were purchased from ATCC. Culture media consisted of a 1∶1 mixture of F12/DMEM supplemented with 0.15 mg/mL Bovine Pituitary extract (VWR), 0.005 mg/mL Porcine Insulin (Sigma), 0.01 mg/mL Human Transferrin (Sigma), 0.1 mM Ethanolamine (Sigma), 0.1 mM phosphoethanolamine (Sigma), 25 nM selenium (Sigma), 500 nM hydrocortisone (Sigma), 0.005 mM forskolin (Sigma), 0.05 μM retinoic acid (Sigma) as previously described [24]. Cells were maintained by passaging no less than 1∶20 in RL-65 media, media changed every other day, with experiments consisting of cells from passages 2–10.

NIH 3T12 fibroblasts were obtained from ATCC. Culture media consisted of DMEM supplemented with 10% Fetal Bovine Serum (Lonza) or Calf Serum (Lonza), 10 IU/mL Penicillin (Cellgro), 10 μg/mL Streptomycin (Cellgro), and 2 mM L-glutamine (Cellgro). Cells grown in FBS were maintained by passaging no more than 1∶5 in 3T12 media, media changed every other day, with experiments consisting of cells from passages 5–10. Cells grown in CS were maintained by passaging no less than 1∶10 in 3T12 media, with media changed every other day, with experiments consisting of cells from passages 3–6.

Costar 0.4 μM 12 mm diameter polyester transwell permeable supports were used for all experiments utilizing transwell setups. Volumes in each chamber were kept at 0.4 mL in apical chamber and 1 mL in basolateral chamber. 2×10^5^ RL-65 epithelial cells or NIH 3T12 fibroblasts were plated per transwell and trans-epithelial resistance (TER) was measured every 24 hours using a Millipore Millicell-ERS. Briefly, the resistance (ohms) of blank transwells with media but no cells was measured and its value subtracted from that of wells with cells at each time point. This number was then multiplied by the effective membrane area (cm^2^) of the transwell membrane to determine TER as ohms/cm^2^.

### Immunofluorescence

#### E-Cadherin and Z0-1

RL-65 cells were plated at a concentration of 2×10^5^/well onto transwell membranes. Confluency was assessed by visual inspection coupled with measuring resistance across the transwell membrane (TER measurements). Confluent monolayers were fixed with 3.7% formaldehyde for 20 minutes at room temperature. Cells were permeabilized using 0.2% Triton X-100 treatment for 10 minutes at room temperature. All samples were incubated in blocking buffer (3% BSA/0.05% Tween) for 1 hour at room temperature before addition of specific antibodies. Specific antibodies [mouse anti - E-cadherin (BD Biosciences) or rabbit anti -Zona Occludens (Invitrogen)] were added at a concentration of 5 ug/mL in blocking buffer, and incubated for 1 hour at room temperature. Finally, secondary Alexa Fluor conjugated- antibodies [Alexa Fluor 488 goat anti-mouse or Alexa Fluor 568 goat anti-rabbit] were added at a concentration of 0.4 μg/mL in blocking buffer and incubated in the dark for 1 hour at room temperature. Samples were mounted with antifade reagent containing DAPI (Invitrogen).

#### YFP

RL-65 cells were plated at a concentration of 2×10^5^ onto 1.7 cm^2^ glass chamberslides (Millipore). After visual confirmation of confluency, cells were infected at a MOI of 0.05 with MHV68-H2bYFP, a virus that expresses the yellow fluorescent protein from infected cells [35]. 120 hours post infection cells were fixed with 3.7% formaldehyde for 20 minutes at room temperature and mounted with antifade reagent containing DAPI (Invitrogen) and visualized for YFP expression.

### Virus Growth Assays

For replication assays in standard 24 well tissue culture plates, RL-65 cells were plated at a concentration of 4×10^5^ cells/well. After visual inspection to confirm full monolayer formation, cells were infected at MOI 0.05 with either MHV68 WT or V.cyclin.stop. Every 24 hours cells plus supernatants were collected. Samples were subjected to freeze/thaw twice to release any intracellular virus. Total virus titer was calculated from plaque assays on NIH 3T12 fibroblasts.

Plaque assays were perfomed on NIH 3T12 fibroblasts. Briefly, 24 hours prior to assay 2×10^5^ NIH 3T12 cells were plated per well onto standard 6 well tissue culture plates. After fibroblasts reached ∼80% confluency samples ready to titer were serially diluted and plated onto fibroblast monolayer. Plates were incubated at 37°C for one hour with gently rocking back and forth to envenly distribute inoculum. After one hour a 2.5% serum/methocelluose solution was overlayed and plates were incubated for approximately one week until plaques were visualized. Plaques were stained with 0.1% crystal violet in 20% methanol.

For growth analyses on polarized RL-65 cell plated on transwells (Costar 0.4 μM 12 mm diameter), cells were plated at a concentration of 2×10^5^ cells per transwell. Monolayer growth was monitored by TER and visual inspection. Confluent monolayers were infected apically at an MOI of 0.05 with either MHV68 WT, V.cyclin.stop, V-cyclin/E133V, or K-cyclin virus. Every 24 hours supernatant from apical and basolateral chamber was collected, or in the case of determining total virus replication cells were scraped off membrane using a sterile pipette tip blunt end and cells plus supernatant from apical and basolateral chamber was collected. Samples were subjected to two rounds of freeze/thaw to release any intracellular virus. Total virus was quantified by plaque assay on NIH 3T12 fibroblasts.

To determine whether any virus becomes trapped in empty transwells, virus was added to apical chamber of transwell setups that contained media only (no cells). WT MHV68 (4×10^5^ pfu) in a volume of 50 μL was added to 350 μL media in apical chambers. Basolateral chambers contained 1 mL of media. Plates were incubated at 37°C and samples from the apical and basolateral chambers were taken at 30 min, 2 hr, and 4 hr after virus addition, and subsequent virus titer determined by plaque assay on NIH 3T12 fibroblasts.

### Generation of MHV68/K-cyclin Virus

PCR was used to clone the KSHV v-cyclin open reading frame (K-cyclin) from the KSHV latently infected BCBL cell line (reference for KSHV cyclin region-GenBank U79416). The K-cyclin coding sequences were cloned using the following primers: 5′-cttgtcgtccttgtagtcatagctgt ccagaat-3′ and 5′-tatatggcaactgccaataacccgccc-3′. PCR was then used to amplify the left and right flanking arms of the MHV68 v-cyclin ORF with designed overlapping sequences matching that of K-cyclin ORF (reference for MHV68 cyclin region-GenBank U97553). Left flanking arm primers: 5′-gggggatcccacacatcaagttatcactttttg-3′ and 5′-gactacaaggacgacggacgacaagttaaaaaat aaatgcc-3′. Right flanking arm primers: 5′-gggcgggttattggcagttgccatata-3′ and 5′-gggtctagaat tgttttcaataaaaaagtg-3′. The flanking arm primers also contained restriction sites BamHI and XbaI for subsequent subcloning. Overlapping-extension PCR was used to generate the final PCR fragment that contained the K-cyclin ORF with flanking sequences from the MHV68 v-cyclin region such that an exact replacement of MHV68 v-cyclin open reading frame with the K-cyclin open reading frame would be generated (i.e., an exact open reading frame replacement from start codon to stop codon was constructed). Chimeric DNA was inserted into pCR-Blunt using BamHI and XbaI and then cloned into the allelic exchange targeting vector pGS284MHV68 using the same restriction sites. MHV68-BAC was generated and bacteria mediated allelic exchange was performed as described previously for MHV68 mutants [17]. After confirming the appropriate recombinants, the BAC vector was removed by passage through Vero-CRE cells as previously described. High titer viral stocks were then generated from infected NIH 3T12 cells as described previously for MHV68 mutants [17].

### Infections and Analysis of Acute Virus Replication in Lungs

C57/Bl6 mice (Jackson laboratories) were placed under isofluorane anesthesia before intranasal inoculation with 20 μL complete media containing 1,000 pfu of MHV68 WT, or MHV68 mutants V-cyclin.stop or K-cyclin. During the course of infection mice were visually monitored for signs of distress and routine care and feeding of animals was carried out by the Emory University Veterinary Staff in Accordance with the Animal Care and Use Guidelines. On days 4 and 9 mice were sacrificed and lungs harvested. Lungs were placed in complete media (1 mL) and frozen at −80°C. Upon thawing, lungs were subjected to mechanical disruption and dilutions of the resulting lysate plated on NIH 3T12 fibroblasts for plaque assay analysis as previously described [17].

### Limiting Dilution Analyses for Assessing ex-vivo MHV68 Reactivation

To determine the frequency of splenocytes capable of reactivating virus from latency, a limiting dilution ex-vivo reactivation assay was performed as previously described [17]. Briefly, splenocytes from infected mice were plated in a two-fold serial dilution fashion (starting with 10^5^ splenocytes per well) on to MEF monolayers in 96-well tissue culture plates. Twenty-four wells were plated per dilution and 12 dilutions were plated per sample. Wells were scored microscopically for cytopathic effect (CPE) at 14–21 days post-explant. Preformed infectious virus was detected by plating parallel samples of mechanically disrupted cells onto MEF monolayers alongside intact cells.

### Statistical Analysis

Statistical data analysis was performed using GraphPad Prism software. Data shown represents one of at least triplicate experiments. Error bars represent standard error mean. Significance was determined by two-tailed, unpaired Student’s t-test with a confidence level of 95%.
